# Study of Preoperative Radiotherapy for Sarcomas of the Extremities with Intensity-Modulation, Image-Guidance and Small Safety-margins (PREMISS)

**DOI:** 10.1186/s12885-015-1633-y

**Published:** 2015-11-16

**Authors:** Barbara Röper, Christine Heinrich, Victoria Kehl, Hans Rechl, Katja Specht, Klaus Wörtler, Andreas Töpfer, Michael Molls, Severin Kampfer, Rüdiger von Eisenharth-Rothe, Stephanie E. Combs

**Affiliations:** 1 Department of Radiation Oncology, Klinikum rechts der Isar, Ismaninger Straße 22, 81675 München, Germany; 2 Institute of Innovative Radiotherapy (iRT), Department of Radiation Sciences (DRS), Helmholtz Zentrum München, Ingolstädter Landstraße 1, 85764 Neuherberg, Germany; 3 Department of Biometrics, Institut für Medizinische Statistik und Epidemiologie, Technische Universität München (TUM), Ismaninger Strasse 22, 81675 München, Germany; 4 Department of Orthopaedic Surgery, Klinikum rechts der Isar, Ismaninger Strasse 22, 81675 München, Germany; 5 Department of Pathology, Klinikum rechts der Isar, Technische Universität München, Ismaninger Strasse 22, 81675 München, Germany; 6 Department of Radiology, Klinikum rechts der Isar, Technische Universität München, Ismaninger Strasse 22, 81675 München, Germany

## Abstract

**Background:**

The aim of the trial is to demonstrate that with the use of modern IMRT/IGRT and reduction of safety margins postoperative wound complications can be reduced.

**Methods/ Design:**

The trial is designed as a prospective, monocentric clinical phase II trial. The treatment is performed with helical IMRT on the Tomotherapy HiArt System© or with RapidArc© IMRT as available. All treatments are performed with 6 MV photons and daily online CT-based IGRT.

A dose of 50 Gy in 2 Gy single fractions (5 fractions per week) is prescribed. Restaging including MRI of the primary tumor site as well as CT of the thorax/abdomen is planned 4 weeks after RT. PET-examinations or any other imaging can be performed as required clinically. In cases of R1 resection, brachytherapy is anticipated in the 2nd postoperative week. Brachytherapy catheters are implanted into the tumor bed depending on the size and location of the lesion. Surgery is planned 5–6 weeks after completion of neoadjuvant RT. All patients are seen for a first follow-up visit 2 weeks after wound healing is completed, thereafter every 3 months during the first 2 years. The endpoints of the study are evaluated in detail during the first (2 weeks) and second (3 months) follow-up. Functional outcome and QOL are documented prior to treatment and at year 1 and 2. Treatment response and efficacy will be scored according to the RECIST 1.1 criteria. A total patient number of 50 with an expected 20 % rate of wound complications were calculated for the study, which translates into a 95 % confidence interval of 10.0-33.7 % for wound complication rate in a binomial distribution.

**Discussion:**

The present study protocol prospectively evaluates the use of IMRT/IGRT for neoadjuvant RT in patients with soft tissue sarcomas of the extremity with the primary endpoint wound complications, which is the major concern with this treatment sequence. Besides complications rates, local control rates and survival rates, as well as QOL, functional outcome and treatment response parameters (imaging and pathology) are part of the protocol. The data of the present PREMISS study will enhance the current literature and support the hypothesis that neoadjuvant RT with IMRT/IGRT offers an excellent risk-benefit ratio in this patient population.

**Trial registration:**

NCT01552239

## Background

Treatment of soft tissue sarcomas of the extremities is a challenge for the interdisciplinary team. In general, radiation therapy (RT) is indicated in stage II and III (not T1a). There are two approaches for RT in this situation, either neoadjuvant or adjuvant. For preoperative RT usually lower doses are applied, and a dose of 50 Gy has been established; in the postoperative setting higher doses between 60-66Gy are required. Thus, preoperative RT seems to be more beneficial in terms of long-term RT-associated side effects such as edema, joint stiffness, nerve lesions or bone fractures. On the other hand, preoperative RT is associated with a higher rates of wound complications after surgery (35 % vs. 17 %). This is more or less independently of the location, i.e. shoulder, upper or lower extremity and of the histology, which can be very heterogeneous including liposarcoma, leiomyosarcoma, undifferentiated sarcoma or synovial sarcoma. Thus, due to the required expertise of all disciplines, patients with such tumors should be treated at a specialized sarcoma unit [1–6], since it has been shown that treatment at a high volume center is associated with a significantly increased survival and better functional outcome [7].

**Fig. 1:**
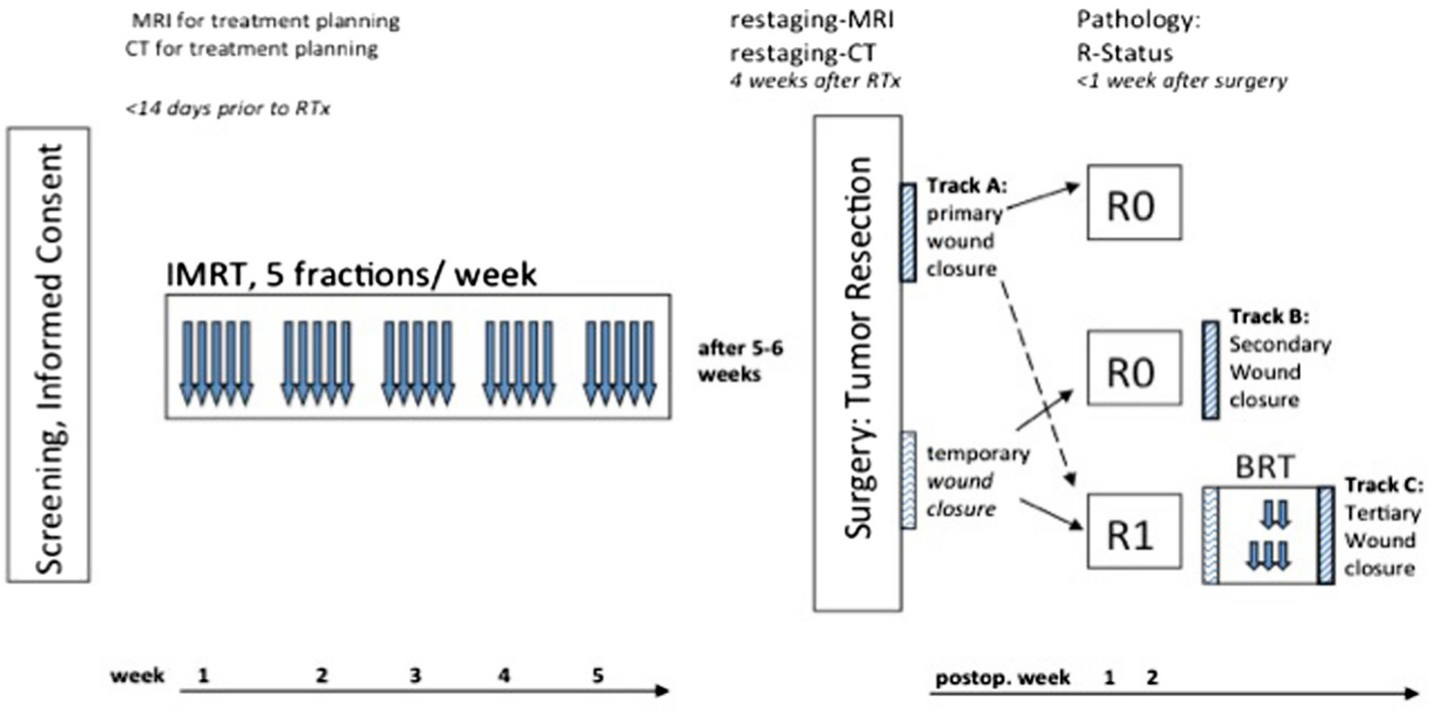
shows the study diagram of the PREMISS Study

Surgery is the mainstay of treatment in sarcomas and should be evaluated in every case. If surgery with a complete removal of the tumor is not possible, RT is a curative alternative; local control rates range between 20–45 % [8]. Generally, function-preserving treatment is a main goal, whereas in the past radical excisions with compartment resections leading to a loss of function, or amputations, were performed regularly; today, function-preserving treatment is anticipated with complete removal where possible, and combination with RT when necessary.

Few randomized studies are available which is mainly due to the low incidence of soft tissue sarcomas: An older trial compared limb amputation with a combination of extremity-conserving resection plus postoperative RT; no difference in disease-free survival and overall survival was observed [9].

Two other trials assessed postoperative RT or interstitial brachytherapy, both studies showed a clear advantage for local control compared to surgery alone [10, 11]. A large number of retrospective analyses have confirmed the positive value of postoperative RT for extremity sarcomas [12–23].

Today, extremity-conserving surgical treatment is possible in 80–95 % of all patients [24–28]. This requires, however, a well-functioning interdisciplinary team consisting of orthopaedic surgeons, radiation oncologists, plastic surgeons, oncologists, pathologists and radiologists [29, 30].

In detail, two main concepts exist for the application of RT: preoperative vs. postoperative. As in other indications such as esophageal, pancreatic or rectal cancer, there are clear arguments in favor of preoperative RT: The treatment volume is generally much smaller, since postoperative changes as well as intraoperatively manipulated tissue including the surgical entry channel and scar do not need to be treated [31]. Compared to postoperative RT, only doses of around 50 Gy are required. The smaller treatment volume together with the lower RT dose result in lower rates of treatment-related side effects. Moreover, tumor as well as normal tissue oxygenation is not impaired due to postoperative scarring; this leads to a higher sensitivity to radiation due to the oxygen enhancement ratio (OER; [23]).

Another factor is the possibility of sterilization around the tumor using preoperative RT, leading to an improved resectability and higher rates of R0-resections [19]. This might be explained by a thickening of the tumor capsule which has been shown in experimental settings. On the other hand, in spite of the clear advantages of preoperative RT, higher rates of surgery-related wound complications have been shown by several groups [21, 32, 33].

A number of comparative analyses between pre- and postoperative RT are currently available in patients with extremity soft tissue sarcomas. A randomized prospective trial by O’Sullivan and colleagues randomized 50 Gy preoperative RT to 66 Gy postoperative RT. In the preoperative group, which consisted of 94 patients, 10 patients received an additional boost up to 16–20 Gy in cases of R1 resections. Initial data showed a slightly improved local control and survival in the preoperative group, however wound complications were 35 % compared to 17 % in the postoperative RT group; function of the extremity was comparable in both groups [13, 18]. Long term data support the beneficial risk-benefit ratio of preoperative RT [14]. In agreement with several retrospective reports lower rates of long-term side effects such as edema, fibrosis, fracture, joint stiffness or nerve toxicity as well as better functional outcome are observed [14, 15, 17, 22, 23, 32]. A meta-analysis including 1098 patients from 5 studies confirmed a higher local control and a higher overall survival of 76 % vs. 67 % for pre-operative RT [34]. A multi-institutional matched-pairs analysis including 821 patients also reported an improved overall survival after preoperative RT [20].

However, it has to be kept in mind that wound complication rates might be higher after preoperative RT with median complication rates of 16–35 %, depending on the series [12, 17–19, 21, 32, 35]. The rate of wound complications is dependent on RT dose, patient age, comorbidities, tumor and resection volume as well as tumor location [18, 21, 32, 36]: For example, patients with wound complications generally have a much larger resection volume than those without complications (919 cm^3^ vs. 456 cm^3^) [33].

Regarding the RT technique, 3D-conformal RT was standard over many years. Modern techniques such as intensity modulated radiotherapy (IMRT) offer improved dose conformality even for long and complex shaped volumes. Treatment planning comparisons analyzing 3D vs. IMRT could show that dose coverage as well as reduction of dose to normal tissue (bone, soft tissue) are better with IMRT [37–39]. Thus, since the implementation of IMRT for the treatment of soft tissue sarcomas, positive results were reported [40]. With helical IMRT as Tomotherapy^©^ dose distributions often are even more conformal, longer volumes can be treated, and the treatment machine offers online MV-CT imaging for position verification. Early reports on Tomotherapy^©^ for sarcomas reported excellent results as well as improved sparing of normal tissue [41–44]. For daily repositioning image-guidance as well as positioning devices are necessary. For extremity tumors, positioning inaccuracies of 1 cm or more have been observed [45]. This can be compensated by adequate treatment volumes as well as IGRT approaches. The improvements of RT techniques enable the radiation oncologist to reduce and adapt treatment volumes. For soft tissue sarcomas, in the past, uncertainties in positioning as well as in target volume definition depending e.g. on insufficient imaging have led to very large safety margins of ≥ 5 cm proximal/distal and 2 cm circumferentially around the visible tumor volume [31, 38, 45, 46]. Since optimized imaging including magnetic resonance imaging (MRI) as well as CT or PET-diagnostics are available, these safety margins can be reduced and IGRT approaches assure high precision of treatment delivery. Results from brachytherapy series have shown that local dose application to the tumor with small margins of 1–2 cm are excellent with local control rates of 79–87 (−100) % [10, 40, 47–50]. The main factor, however, is exact definition of the target volume and precise dose delivery. Since complication rates are dependent on the irradiated volume, the rationale for smaller safety margins is an optimization of the risk-benefit ratio [33, 51]; initial clinical data on IMRT for soft tissue sarcomas of the extremity have shown local control of 96 % at 3 years with small margins of 2 cm [52]. Intraoperative Radiotherapy (IORT) or brachytherapy can offer an enhanced therapeutic ratio: With both techniques local dose escalations directly to the target tissue are possible, without irradiation of large areas of normal tissue.

For neoadjuvant RT, doses of 50 Gy have been established, however, in some cases incomplete tumor resection with R1 margins requires individualized approaches. It has been shown that local dose escalation as a boost treatment up to 16–20 Gy with conventional fractionation can be performed, however, series from the literature show controversial results [18, 53].

Combination of percutaneous RT (40–50 Gy) and a brachytherapy boost (15–32 Gy) has been reported to be superior to percutaneous RT or brachytherapy alone [25, 49, 54–58]. Thus, combination treatments are considered as optimal for soft tissue sarcomas with positive resection margins [29, 59].

### Rationale for the PREMISS study

Since the techniques of RT have been improved over the last decades, novel concepts for the treatment of soft tissue sarcomas are possible. This includes IGRT approaches with reduced safety margins to improve the therapeutic window in terms of reduction of long-term side effects. Since theoretical advantages of IMRT/IGRT in this patient population have been shown, and initial clinical data confirm this hypothesis, a prospective evaluation of preoperative IMRT/IGRT is necessary. Thus, reduction of safety margins around the visible tumor on MRI of 3 cm longitudinally and 1.5 cm circumferentially is possible based on previously published data [16]. For optimal surgical treatment, three treatment paths are defined for optimal surgical results.

Since in < 30 % of all patients treated with function and extremity preserving RT a R1 resection is present, local dose escalation in analogy to the randomized trial by O’Sullivan is part of this PREMISS trial.

The aim of the trial is to demonstrate that using modern IMRT/IGT and reduction of safety margins, postoperative wound complications can be reduced.

### Endpoints of the study

The primary endpoint is the hypothesis that with preoperative IMRT/IRGT using small safety margins in combination with local dose escalation with brachytherapy in the R1 situation a wound complication rate of 20 % can be achieved.

Thus, the rate of wound complications up to 90 days after surgery is scored. Wound complications are defined as

1. any surgery for wound treatment requiring local or general anesthesia including debridement, operative drainage, secondary or repeated wound closure including rotational plastic, any free tissue transfer or skin transplantations exceeding the procedures included into the protocol
2. invasive procedures without anesthesia, e.g. 3 x aspiration of seroma
3. in-patient wound treatment e.g. intravenous antibiotics
4. <90 days treatments with wound dressing materials

Secondary endpoints of the study are determination of R0-resections, local control, metastases-free survival, overall survival, as well as acute and late toxicities of RT. This includes rates of extremity preservation, function of the extremity as well as quality of life (QOL).

## Study design

The trial is designed as a prospective, monocentric clinical phase II trial. The study design is depicted in Fig. 1.

### Treatment planning for preoperative RT

The extremity will be positioned in a stable and reproducible position using vacuum mats or mask material as necessary. For the planning CT all scars are to be marked with wire. If necessary, bolus material is added and fixed in a reproducible manner.

Treatment planning is based on a CT with 3 mm slice thickness, including the visible tumor and the adjacent joint regions, at least 20 cm beyond the visible tumor. Fusion with MRI is performed within the treatment planning system. MR imaging should include coronal T2 stir, axial T2 with and without contrast, T1 stir with contrast enhancement.

### Target Volume definition

The treatment volumes are defined on the planning CT including the following volumes:

- *primary tumor (PT):* macroscopic tumor on contrast-enhanced MRI
- *gross tumor volume (GTV):* PT plus surrounding pseudo capsule, i.e. edema and edematous changes tissue including tumor cell contamination
- *clinical target volume (CTV):* GTV plus safety margins – 1 cm in lateral and ventro-dorsal direction, as well as 2.5 cm in proximal-distal direction. Natural borders are respected, i.e. skin or non-infiltrated bony structures as well as uninvolved compartments.
- *planning target volume (PTV):* CTV plus a circumferential safety margin of 0.5 cm.

Additionally, all relevant organs at risk (OAR) and normal tissue structures are contoured.

### Treatment technique and dose prescription

The treatment is performed with helical IMRT on the Tomotherapy HiArt System^©^ or with RapidArc^©^ IMRT as available. All treatments are performed with 6MV-photons and daily online CT-based IGRT.

A dose of 50 Gy in 2 Gy single fractions (5 fractions per week) is prescribed to the median in accordance with ICRU 83, with D_50%_ = 50.0 Gy. At least 95 % of the PTV must receive 95 % of the prescribed dose, i.e. D_95%_ > 47.5 Gy.

### Surgical treatment

Surgery is planned 5–6 weeks after completion of neoadjuvant RT. Re-staging including MRI as well as CT of the thorax is planned 4 weeks after RT. PET-examinations or any other imaging can be performed as required clinically.

If possible, the tumor will be resected surrounded by a layer of healthy tissue „en bloc“ in terms of an oncological radical resection ("wide/radical resection"). The resection entry channel from the diagnostic biopsy has to be included completely into the resection including the skin. An incomplete or reductive surgery is to be avoided. Reconstructive surgery for function preservation is anticipated. Curative approaches are the primary goal in situations when function and extremity preservation is not feasible.

The resection specimen must be clipped and marked so that correct anatomical reconstruction and correlation with imaging is possible. The surgeon will clip areas of potential incomplete resection on the resected tissue as well as in the tumor bed.

In cases of lymph node involvement on re-staging examinations in the area of the lymphatic spread of the tumor lymphadenectomy is performed. In cases of lung metastases after neoadjuvant RT or at the time of re-staging local control is still a priority, thus tumor resection is performed. Thereafter, any other measures necessary are taken, such as resection of lung lesions, chemotherapy, RT or other.

In cases of initial complete resection of the tumor direct closure of the wound is performed (Track A). If intraoperative rupture of the tumor occurs or if indication for hypobaric treatment or plastic surgery is present, vacuseal will be brought into the resection cavity and the wound is closed secondarily (Track B and C).

Within 5 days after tumor resection results of the pathological evaluation are available.

If the tumor is resected completely (R0), vacuseal is removed and the wound is closed (track B), if necessary with plastic surgery. If pathology reveals R1 status, secondary resection should be evaluated. If this is not possible with a function-preserving approach, local brachytherapy treatment in the resection cavity is performed. Thereafter, the wound is closed.

### Pathology assessment

For precise pathological evaluation precise orientation of the resected specimen is necessary, thus, it is recommended that a pathologist is present at the time of tumor resection. Classification of tumor resection margins is of high importance since the indication for local boost dose escalation is dependent on this result. Boost treatment should be performed on day 6–8 after resection. Pathological classification should therefore be performed within 5 days after surgery and resection margins (R0, R1, Rx) have to be communicated to the orthopaedic surgeon and the radiation oncologist.

Besides resection margins, tumor grading as well as further immunohistochemical stainings for exact pathological diagnosis will be performed. The tumor will be measured in all dimensions (in cm). Response to RT according to the established pathological protocol for osteosarcomas according to Salzer-Kuntschik will be evaluated [60]. Vital tumor cells will be evaluated as established also for osteosarcomas [61].

### Local dose escalation

In cases of R1 resection brachytherapy is anticipated in the 2. postoperative week. Brachytherapy catheters are implanted into the tumor bed depending on the size and location of the lesion.

Treatment planning is based on 3D-CT imaging with 3 mm slice thickness as well as the most recent MRI available.

The CTV_BRT_ for the brachytherapy application includes the R1-area plus a 5 mm safety margin, or a boost the complete resection cavity plus 5 mm safety margin. No additional PTV_BRT_ is added since the catheters are implanted directly into the target area.

Brachytherapy is performed using Iridium-192 High-Dose Rate (HDR)-afterloading. A dose of 12–15 Gy with 3 Gy single doses and 2 fractions per day (≥6 h between fractions) with D_90%_ for the CTV/PTV_BRT_ is applied.

### Further evaluations

To characterize the effectivity of neoadjuvant IMRT/IGRT for extremity sarcomas, the following evaluations will be performed:

- comparison of “conventional safety margins” and reduced safety margins within the protocols on treatment planning comparisons and calculation of dose reduction to normal tissue
- evaluation of tumor response on MRT as well as statement on resectability of the operating orthopaedic surgeon prior to resection based on imaging only
- histopathological characterization of the tumor and tumor response to treatment
- correlation of tumor response with outcome and prognosis.

### Inclusion criteria:

- histologically confirmed and imaging defined soft tissue sarcoma of the extremities
- AJCC-Stage II or III (without T1a-tumos, no N1)
- primary or recurrent tumor
- after biopsy or previous R2 resection
- based on imaging, „primary resectability“ or potential resectability after neoadjuvant RT must be present
- age ≥ 18 years
- ECOG Performance Status 0–2
- informed consent

### Main exclusion criteria

- extraskeletal tumors of the Ewing-/PNET-group
- extraskeletal osteo- or chondrosarcoma
- aggressive fibromatosis (desmoid tumors)
- dermatofibrosarcoma protuberans
- presence of lymph node metastases (N1) or distant metastases (M1)
- expected survival < 1 year
- pregnancy, adequate contraception until 3 months after RT
- severe comorbidities impairing study treatment
- severe wound infections or recurrent skin infections
- known positive HIV-Status
- surgery of the primary tumor or chemotherapy within the last two weeks prior to study treatment
- persistent toxicity of other tumor treatments in the treatment region
- simultaneous chemotherapy, targeted therapy or experimental tumor therapy
- previous RT in the treatment region
- medication with steroids or immuno-suppressants

### Follow-up

All patients are seen for a first follow-up visit 2 weeks after wound healing is completed, thereafter every 3 months during the first 2 years. The endpoints of the study are evaluated in detail during the first (2 weeks) and second (3 months) follow-up.

Functional outcome and QOL are documented prior to treatment and at year 1 and 2.

Treatment response and efficacy will be scored according to the RECIST 1.1 criteria.

### Sample size calculation

A total patient number of 50 with an expected 20 % rate of wound complications was calculated for the study; the intent to treat (ITT) collective includes all patients included into the trial which signed informed consent and were allotted a patient study number. The per protocol collective (PP) includes only those patients, whose study treatment was applied completely without any severe protocol deviations.

Analysis for the primary and secondary endpoints are performed on the ITT collective, and re-evaluated in the PP group. The primary endpoint is the rate of wound complications 3 months after wound closure, including the 95 % confidence interval. The secondary endpoints are analyzed with an explorative approach. The rate of wound complications per treatment track is evaluated as means including the 95 % confidence interval. Survival rates are determined using the Kaplan-Meier Method.

## Discussion

Neoadjuvant RT is an established treatment approach for extremity sarcomas, showing beneficial results compared to postoperative treatment. A major downside are increased rates of wound complications compared to postoperative RT. However, with modern RT approaches such as IMRT and IGRT, treatment precision is optimized with daily image guidance.

In the past, large safety margins were necessary to provide optimal oncological treatment, however, these safety margins most probably also contributed to the high rates of side effects since large amounts of normal tissue were exposed to RT.

The use of modern techniques enables the radiation oncologist to deliver precise RT doses, therefore margins around the tumor can be reduced which leads to sparing of normal tissue.

The present study protocol prospectively evaluates the use of IMRT/IGRT as neoadjuvant RT in patients with soft tissue sarcomas of the extremity with the primary endpoint wound complications, which is the major concern with this treatment sequence. Besides complications rates, local control rates and survival rates, as well as QOL and functional outcome as well as treatment response parameters (imaging and pathology) are part of the protocol. The data of the present PREMISS study will enhance the current literature and support the hypothesis that neoadjuvant RT with IMRT/IGRT offer an excellent risk-benefit ratio in this patient population.

## Abbreviations

BRT: Brachytherapy
CT: Computer Tomography
CTV: Clinical Target Volume
GTV: Gross Tumor Volume
IGRT: Image Guided Radiotherapy
IMRT: Intensity Modulated Radiotherapy
IORT: Intraoperative Radiotherapy
MRI: Magnetic Resonance Imaging
PTV: Planning Target Volume
